# A framework checklist for implementing bedside electronic transfusion systems

**DOI:** 10.1111/tme.70042

**Published:** 2025-11-14

**Authors:** Josephine McCullagh, Suzanne Makki, Kirsty Hancock, Catherine Booth, Louise Bowles, Ollie Djurdjevic, Karen Farrar, Claudio Geraci, Sara Hammond, Helinor Mcaleese, Michael F. Murphy, Florence Oyekan, Laura Green

**Affiliations:** ^1^ Barts Health NHS Trust London UK; ^2^ NHS Blood and Transplant London UK; ^3^ Blizard Institute Qeen Mary University of London London UK; ^4^ Bolton NHS Foundation Trust Bolton UK; ^5^ Oxford University Hospitals NHS Foundation Trust Oxford UK; ^6^ University of Oxford Oxford UK

## Abstract

**Background:**

Every time a unit of blood is given to a patient, it is essential that all steps in the transfusion pathway are executed correctly to ensure that the right blood is transfused to the right patient. Bedside transfusion checks at the point of sampling for compatibility testing, sample labelling and blood administration are an essential part of the delivery of safe transfusion and avoidance of the wrong blood being given, which can have serious consequences. Implementation of bedside electronic transfusion systems that use barcode matching of patients' wristbands and blood units is now recommended as the best practice to ensure patients' safety in transfusion. However, there is limited information in the literature to guide hospitals on what aspects they should consider when introducing a bedside electronic transfusion system.

**Aims and Methods:**

This paper aims to support hospitals considering implementing a bedside electronic transfusion system by providing a comprehensive checklist addressing planning, stakeholder coordination, device integration, and compliance with national standards and safety requirements.

**Results:**

The checklist is based on the experiences of two NHS Trusts in the UK and aims to provide organisations with a resource to support this change and reduce avoidable delays.

## 1 BACKGROUND

Blood donated for transfusion is a scarce resource and is a life‐saving treatment for many patients. Every time a unit of blood is given to a patient, it is essential that all steps in the transfusion pathway are executed correctly to ensure that the right blood is transfused to the right patient. These steps include collection of the blood sample for compatibility testing, laboratory procedures for providing compatible blood, the collection of blood from the laboratory or remote blood fridge and the bedside administration checks of blood. Bedside checks at the point of sampling for compatibility testing, sample labelling and blood administration are an essential part of the delivery of safe transfusion and avoidance of the wrong blood being given, which can have serious consequences.^1^

The use of manual bedside checks has led to the introduction of several mitigation steps for safe transfusion. These include: (1) requiring two independent samples to confirm a patient's group prior to the issue of group‐specific components; (2) transfusion laboratories rejecting any mislabelled blood samples with zero tolerance for any mislabelling; and (3) pre‐transfusion checks being undertaken by two members of staff. While these steps have improved transfusion safety, they have not prevented errors and occasional wrong transfusions.^1^ Furthermore, these measures are inefficient because they duplicate work throughout the transfusion pathway. In addition to manual safety measures, Bedside Electronic Transfusion Systems (BETS) uses barcode matching of patients' wristbands and blood units making it easy for staff to ensure that patients receive the correct blood unit, they also streamline the process by requiring one clinician rather than two to perform checks prior to blood administration and potentially removing the need for a confirmatory group‐check sample.^2^

However, there is limited information in the literature to guide hospitals on what aspects they should consider when introducing a BETS. Hence, this paper aims to provide a checklist tool on the implementation of BETS, something that we would have benefitted from had it been available prior to starting this journey. The checklist will support hospitals considering implementing a BETS by addressing planning, stakeholder coordination, device integration, and compliance with national standards and safety requirements. The checklist (Table 1) is based on the experiences of two NHS Trusts in the UK and aims to provide organisations with a resource to support this change and reduce avoidable delays.

**TABLE 1:** Checklist for implementing bedside electronic transfusion systems in hospitals.

Stage	Action item	Status
Process mapping	Conduct detailed process mapping to consider Number of devices Compatibility sample process (inpatient and outpatient) Blood collection Compatibility labels Blood products Pre‐administration checklists Patient wristbands Fate messaging Emergency transfusions Interface requirements for LIMS/EPR and update reflex messages to align with new process Visit other sites who are already using these systems to ensure optimal configuration of devices	–
Business case	Define the rationale and objectives for BETS implementation	–
	Outline safety and compliance benefits	–
	Quantify anticipated productivity and efficiency gains	–
	Estimate long‐term cost savings	–
	Detail resource requirements for implementation and maintenance	–
	Include a risk assessment and mitigation plan	–
	Incorporate stakeholder feedback and support	–
Stakeholder engagement	Identify and engage with key stakeholders	–
	Establish regular communication channels.	–
	Utilise clinical champions and project ambassadors	–
Governance and project structure	Establish a project board and working group. Assign project roles	–
	Develop a risk register and populate with potential hazards, mitigation plans, and action owners	–
	Schedule regular progress meetings with governance bodies and stakeholders	–
Risk and safety management	Develop a comprehensive clinical risk management plan	–
	Conduct hazard identification workshops to document potential risks and control measures.	–
	Implement and document risk control measures	–
	Prepare and train for contingency plans	–
Technical pre‐requisites	Test compatibility with existing IT systems	–
	Ensure secure network connectivity and coverage	–
	Agree ongoing technical support and maintenance	–
	Coordinate with IT to address compatibility and infrastructure needs	–
	Update all laboratory and clinical documentation	–
	Determine how and when the devices will be used: Blood components/products Elective/emergency Inpatient/outpatient Pilot the system, evaluate lessons learned and update documents accordingly prior to wider rollout	–
Training and user support	Develop training materials, including practical, simulation‐based sessions, and video demonstrations	–
	Incorporate BETS training into existing training programmes and competency documents	–
	Establish cascade trainers	–
	Track training completion and competency	–
	Put in place a clear pathway for staff to report problems/errors with devices	–
	Create an inventory for handing over and replacing devices	–
	Establish an in‐house team that will support the long‐term maintenance of devices (e.g. overseeing the inventory and the continuous use of devices)	–
	Assign responsibilities for regular monitoring and testing of devices on the wards	–
	Agree who is responsible for replacing equipment in hospital	–
Evaluation	Establish key performance indicators (KPIs)	–
	Conduct regular audits and compliance checks	–
	Gather user feedback for system refinement	–
	Complete the handover form to move to business as usual	–

## 2 PROCESS MAPPING

Comprehensive process mapping is a critical first step in the implementation of a BETS; we recommend the use of the 10‐step framework reported by the national haemovigilance scheme (Serious Hazards of Transfusion, SHOT) and for each hospital to adopt the mapping according to its needs.^3^ It provides a thorough understanding of existing transfusion pathways in the hospital, allowing teams to identify issues that need to be addressed by the new system in different clinical areas. This detailed assessment is essential to accurately determine the resources needed, build a strong business case, and ensure the correct specifications are included in the user requirement specification for procurement.

Mapping the processes is especially important in multi‐site trusts, as resource needs and workflows may differ. Consider the following questions to scope the work relating to the implementation:

- How many Personal Digital Assistant devices and printers (including spares) will be required for each area?
- Are blood samples for compatibility testing requested electronically or by paper? Will this change after implementing BETS?
- What are the requirements for collecting blood components from the laboratory or remote fridges, and will these need to change with BETS integration?
- Are the scannable barcodes in the blood compatibility labels compatible with BETS or will the formatting need to change? Are new printers required?
- Will the BETS be used for blood products (e.g. albumin) as well as blood components?
- Will the pre‐administration checklist policy, including positive patient identification, need to change with BETS?
- Do patient wristbands contain a 2D scannable barcode that is compatible with the BETS? Will this need to be updated?
- Does the hospital have processes for making it easy to print barcoded wristbands in all areas where transfusion activities occur, and is this the same for elective and emergency transfusion? Can emergency departments provide printed barcoded wristbands in urgent clinical scenarios?
- Do the devices need to interface with the electronic patient record (EPR) or the laboratory information management system (LIMS) or both for fating of the blood units?

In addition:

- Consider what is already recorded in real time in the EPR by the patient's bedside (e.g. observations like blood pressure) to avoid duplication for staff.
- Identify interoperability limitations between the different systems and plan around it.
- Consider integration of checklists (e.g. TACO or consent) to device usage.

Early coordination with the hospital's information technology (IT) team, LIMS providers and suppliers helps avoid issues and ensures that the system integrates seamlessly with the hospital's current infrastructure. This proactive approach mitigates the risk of delays and technical challenges, creating a stable foundation for the BETS. Consider visiting other sites that are already using BETS (ideally more than one) to learn from what works well and how best to design your system.

## 3 BUSINESS CASE DEVELOPMENT

Developing a comprehensive business case is essential for securing the necessary support and resources to implement BETS effectively. A well‐structured business case outlines the clinical and operational needs for an electronic transfusion system, highlighting safety improvements, detailing the anticipated benefits on productivity and service quality and emphasising efficiency savings because of the removal of second staff from blood administration checks. By clearly documenting these elements, the business case demonstrates the value of BETS and establishes a persuasive case for investment, aligning stakeholders and leadership with the project's objectives. The business case must:

- Define the rationale and objectives for BETS implementation, emphasising patient safety, reducing human errors and improving regulatory compliance particularly for traceability in transfusion processes.^4^
- Quantify anticipated productivity and efficiency gains by providing concrete evidence of BETS's operational value, optimising resources, reducing workload, and improving staff productivity.
- Analyse long‐term cost savings and return on investment to strengthen the financial justification. Data from process mapping should provide an estimate of potential gains through reduction in sample rejection rates and improved staff efficiency.
- Detail resource requirements for short‐ and long‐term implementation and maintenance of the system to allocate resources effectively and confirm that the organisation is prepared to support BETS sustainably.
- Incorporate stakeholder feedback and support. This endorsement builds a broader base of support and reinforces the relevance of BETS across departments.

## 4 GOVERNANCE

Prior to the project starting, a separate governance structure should be convened to oversee the implementation of the new system, with key stakeholders being represented in relevant committees to allow for optimal communication and support between teams. The dedicated project governance should have a separate project board (represented by senior leaders in the hospital, including transfusion) to provide strategic oversight to the projects, including expert advice and guidance during the full project cycle, monitoring progress, holding the project working group accountable to agreed milestones, and guiding against hospital‐wide risks, with ultimate responsibility being held by the chair and the chief executive of the hospital.

A project working group should have a dedicated project manager and key members of the transfusion team (transfusion practitioners, laboratory managers, pathology IT specialists, and quality team). The main roles of the group are to produce the project plan, track risks and issues, and assist with mitigation of risks. The working group should provide a forum for learning and sharing to wider teams and promote the project across the whole hospital. The project working group should report to the project board and hospital transfusion committees.

## 5 STAKEHOLDER ENGAGEMENT

Effective stakeholder engagement and clear communication are fundamental to the successful implementation of BETS. Engaging key stakeholders early helps to build a coalition of support across departments, ensuring that the system aligns with the needs and expectations of all parties involved in the transfusion process. Additionally, transparent communication throughout the project fosters a sense of ownership among staff, reduces resistance to change, and addresses potential concerns proactively.

Key stakeholders should include clinical leads from all areas where the new system will be implemented (e.g. surgery, trauma, paediatric etc.), laboratory senior managers, IT professionals, nurses, procurement teams, the education team and patients. Each of these groups brings unique insights and expertise that are critical to customising the BETS system to meet hospital needs.

### 5.1 Establish regular communication

Regular communications ensure that all staff members understand the BETS implementation timeline, their roles, and how the new system will affect their daily tasks. They also provide an avenue for stakeholders to ask questions, raise concerns, and contribute feedback, fostering a sense of inclusion and transparency.

Promoting the launch of the system and its intended benefits for staff and patients can create a positive feeling, raise awareness, and increase the chance of a successful roll‐out. Multiple communication channels should be used, such as an intranet page, a dedicated launch event, departmental meetings/huddles, audit days, and transfusion committees.

Clinical champions and project ambassadors can act as advocates for the system and help bridge the gap between the project team and front‐line staff, enhancing acceptance for the new system and easing transitions by addressing concerns in real time. These champions will help communicate the benefits of BETS, assist with troubleshooting, and gather feedback to support the implementation and ongoing use of BETS.

## 6 RISK MANAGEMENT

Risk management and safety assurance are essential components in the implementation of BETS. Given that transfusion errors can have serious consequences, managing potential risks associated with BETS implementation is critical to protecting patient safety. An effective risk management strategy mitigates against device malfunctions, data entry errors, or operational disruptions, ensuring that the system operates as intended without compromising the quality of care.

### 6.1 Develop a comprehensive clinical risk management plan

A clear risk management plan ensures compliance with national IT safety standards,^5^ identifies potential hazards and defines control measures for each risk. Identify potential hazards associated with BETS implementation, such as technical issues, user resistance, device malfunction or network connectivity challenges. Evaluate the risks and for each risk, define and document a mitigation plan outlining measures to reduce the risk to an acceptable level. Include the plan within the hospital's risk management system.

### 6.2 Contingency plans

Contingency plans protect patient safety by ensuring that transfusions can proceed safely even if BETS is temporarily unavailable (e.g. network failure). Providing alternative workflows for critical tasks minimizes delays and helps maintain confidence in the safety and reliability of the transfusion process under any circumstance. Training should continue to include manual methods as an alternative procedure to ensure continuity of safe transfusion practices.

## 7 TECHNICAL PRE‐REQUISITES

A thorough understanding of the technical prerequisites is essential to ensure that BETS can be seamlessly integrated into the existing transfusion workflow. Addressing these foundational requirements helps enable a smooth implementation.

### 7.1 Determine the scope of device usage

Determining the clinical settings where the BETS devices will be used is essential to ensure that these are configured correctly. Considerations should be made around whether the devices will be used for:

- Blood components only or blood components and products (such as albumin, anti‐D).
- Elective and emergency transfusions – for emergency transfusion workflows may need to be redesigned to allow the use of BETS.
- Inpatient and/or outpatient compatibility testing – patients wearing wristbands will be the main consideration when introducing BETS in the outpatient setting.

### 7.2 Ensure secure network connectivity

Reliable network connectivity is essential for real‐time data transmission and system responsiveness. Strong connectivity across the hospital reduces delays, prevents data loss, and maintains the integrity of transfusion records. Confirm that all clinical areas, including the laboratory, have strong, secure connectivity for BETS devices. Work with IT to address any areas with weak signal or connectivity issues.

### 7.3 Test compatibility with existing IT systems

IT compatibility testing minimises the risk of technical failures that could disrupt service or compromise data accuracy. The following should be checked:

- Verify that the devices can transfer data accurately and securely to other hospital IT systems without lag or data loss.
- Determine which data variables will be transferred to LIMS or EPR (e.g. component fate for traceability, volumes, observations, reactions). The selected variables could streamline data collection for internal and national audits.
- Ensure that patient wristbands and blood compatibility labels contain the relevant barcodes compatible with BETS.
- List all laboratory and clinical documentation that will require updating and ensure the new process aligns with other processes in the hospital.

Pilot the system in an area of high transfusion activity (e.g. haematology day unit). Allow sufficient time for running the pilot and evaluating lessons learned before proceeding with the wider rollout.

### 7.4 Ongoing technical support and maintenance

Access to consistent technical support helps maintain device performance and user satisfaction. Regular maintenance ensures devices function as intended, preventing unexpected downtime. A service‐level agreement (SLA) should be established with the device supplier outlining the support for troubleshooting, regular maintenance, software updates, and device replacement if needed. Involvement from the IT department is essential in understanding software updates and requirements. Adding the BETS devices to the trust's mobile device management (MDM) system could be an option.

Put in place a clear pathway for staff to report problems/errors with devices to ensure these are corrected promptly to avoid delays for patients but also for staff becoming disillusioned with the system or developing workarounds.

The project and transfusion teams should establish in advance the in‐house team that will support the long‐term maintenance of these devices, including troubleshooting problems with hardware and software, operating systems upgrades and overseeing the inventory and the continuous use of devices.

## 8 TRAINING AND USER SUPPORT

Comprehensive training and strong user support are essential for the successful adoption and effective use of the BETS system. Training ensures that staff at all levels understand how to use the new technology, follow safe transfusion protocols, and respond correctly to any issues that may arise. Effective user support helps staff troubleshoot issues promptly and maintains confidence in the system. Together, these elements are critical for ensuring that BETS is consistently used as intended, minimizing the risk of errors and maximizing safety and efficiency.

### 8.1 Develop clear training materials

**FIGURE 1:**
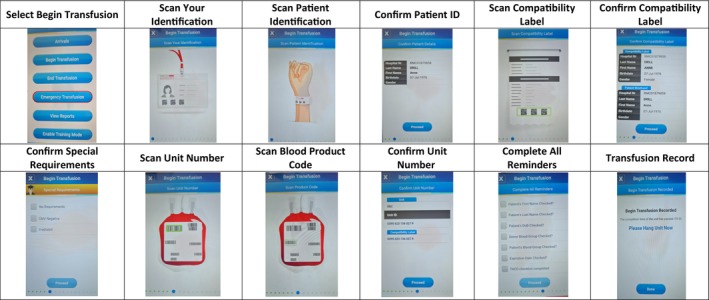
Training material for administering blood components using BloodTrack devices.

High staff turnover or the presence of temporary staff can impact system reliability if they are not properly trained. Providing easily accessible resources ensures that all users can quickly learn how to use devices safely and effectively (see Figure 1 as an example). Create comprehensive, easy‐to‐follow training resources tailored to the specific needs of different user groups, including nurses, healthcare assistants and doctors. Include a combination of formats, such as step‐by‐step guides, video tutorials, and hands‐on practice sessions, to cater to various learning styles. Create and distribute resources to aid with technical issues, frequently asked questions, and troubleshooting assistance. Incorporate competency assessments to verify staff understanding.

### 8.2 Establish cascade trainers

Immediate support reduces frustration and helps maintain workflow efficiency, especially during the initial rollout when users are still acclimatising to the system. Practice educators or digital nurses should be approached and trained as cascade trainers. Cascade trainers can enhance training by providing accessible, on‐the‐ground support, addressing issues in real time, and promoting system adoption. They also provide a key point of contact in each clinical area.

Work with the cascades trainer to assign responsibility for regularly monitoring and testing devices in the clinical areas and agree on the pathway for how to manage or replace lost or faulty equipment.

### 8.3 Track training compliance

Tracking completion and competency provides evidence that all users are adequately trained, which is crucial for releasing devices to the ward. Integrating BETS training into routine training/competency assessments ensures that all staff, including new starters, are equipped with the knowledge they need to use the devices safely and effectively. This approach builds long‐term familiarity with the system, embedding BETS proficiency as part of standard transfusion training.

Use a training management system to monitor staff completion of initial training and refresher sessions. Regularly monitoring training compliance can help reduce variability in system use, supporting a consistent, standardized approach across departments.

## 9 EVALUATION AND CONTINUOUS IMPROVEMENT

Evaluation and continuous improvement are essential for ensuring that BETS meets its objectives in terms of safety, efficiency, and user satisfaction. Continuous evaluation identifies areas for refinement, addresses unforeseen issues, and captures feedback for ongoing system optimisation.

### 9.1 Establish key performance indicators

Key performance indicators (KPIs) provide measurable benchmarks that help assess the impact of BETS on patient safety, workflow efficiency, and overall success. Tracking these indicators allows the hospital to quantify improvements and identify any areas where additional support or adjustments are needed. Define and monitor KPIs relevant to BETS, such as use of devices, reduction in compatibility sample rejections, traceability, and compliance rates with BETS procedures. Regularly review these metrics to evaluate system performance.

### 9.2 Regular audits

Audits, including observation of device use in practice, ensure that BETS protocols are consistently followed and highlight any deviations that could compromise patient safety. Compliance checks are also an opportunity to identify training gaps, refine processes, and strengthen adherence to safety protocols. Perform a baseline audit pre‐implementation to quantify benefits of the system and implement routine audits to review compliance with BETS protocols, assess data accuracy, and monitor system usage.

### 9.3 User feedback

Feedback from staff and patients provides valuable insights into the system's usability, functionality, and any areas where improvements may be needed. By involving users and patients in the evaluation process, the hospital demonstrates a commitment to addressing concerns and optimising the system based on real‐world experience. Use surveys, feedback forms and focus groups to capture insights from end‐users. Having a presence on the wards is an effective way of ensuring feedback, as users may not have time to respond to emails or forms. This also allows concerns raised to be addressed rapidly and users reassured that their feedback is being considered.

In conclusion, a structured and systematic approach to planning, executing, and providing ongoing operational support can help with a successful implementation of BETS. This not only demonstrates commitment to improvement in service by promoting a culture of excellence, but it also aligns with best practices and national recommendations, including those from the Infected Blood Inquiry^6^ and haemovigilance schemes.^1^

## AUTHOR CONTRIBUTIONS

Laura Green, Josephine McCullagh, Suzanne Makki, and Kirsty Hancock designed the study, reviewed the manuscript, and made all subsequent revisions. All other authors contributed to the writing of the manuscript. All authors read and approved the final manuscript.

## FUNDING INFORMATION

This work has been supported by a grant from Barts Charity and a grant from NHS Charities Together (through Barts Charity).

## CONFLICT OF INTEREST STATEMENT

Mike Murphy has received consultancy fees from Haemonetics for speaking at webinars and other events. All other authors declare no conflict of interest.

## Data Availability

The data that support the findings of this study are available on request from the corresponding author. The data are not publicly available due to privacy or ethical restrictions.
